# The Central Medical Society of Illinois

**Published:** 1851-07

**Authors:** 


ARTICLE IV.

*The Central Medical Society of Illinois.*

This Society held its regular meeting at Middletown, Logan Co., Ill's., May 27th 1851.

A paper was read before the Society, containing "Observations on Cerebro-Arachnitis or Meningitis, as it occurred in Clark, and Menard Cos., Ill's." After which a short address was read by Dr. Chamberlain, which papers were ordered to be published in the "North-Western Medical and Surgical Journal."

A Preamble and resolution was then adopted, to wit:

*Whereas,* in consequence of high waters, and other interfering circumstances, the Physicians of the Central Medical Society were prevented from attending the regular annual meeting of said society, at which its annual election of officers should have been held—Therefore, Resolved, that said election be deferred until the first regular meeting of the Society, on the second Wednesday in August next.

It was also further Resolved, That the Regular meetings of this Society for the current year shall take place as follows:—On the second Wednesday in August, and the second Wednesday in November, 1851; the second Wednesday in February, and the second Wednesday in May, 1852.

Resolved that the times for holding the Regular Meetings of this Society, be published in the Springfield weekly papers, and the proceedings thereof in the North Western Medical and surgical Journal.

On Motion, Dr. Chamberlain was appointed a Delegate to the State Medical Society, to be held in the city of Peoria, on the first Tuesday in June next.

Adjourned to meet on the second Wednesday in August next.

